# Supplementary material for: The Vasoactive Role of Perivascular Adipose Tissue and the Sulfide Signaling Pathway in a Nonobese Model of Metabolic Syndrome
Source: Biomolecules. 2021 Jan 15;11(1):108. doi: 10.3390/biom11010108 (PMC7829844; doi:10.3390/biom11010108)
Supplement: Supplementary file 1 [file biomolecules-11-00108-s001.pdf]

Figure S1: Transmission electron photomicrographs of the arcus aortae in HTG rats – cross section of the intimal part

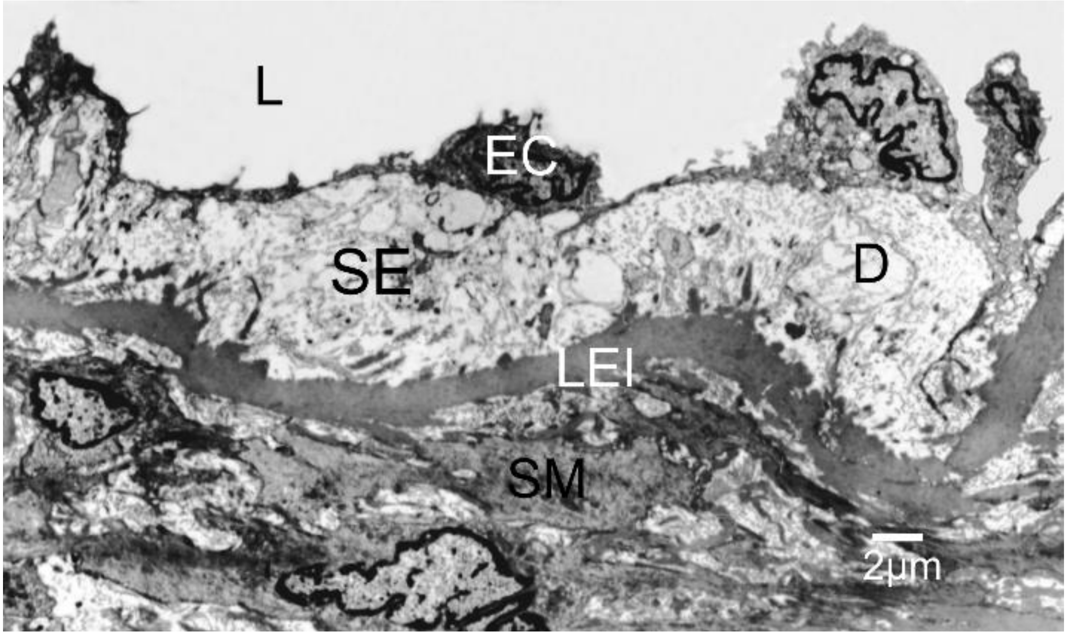

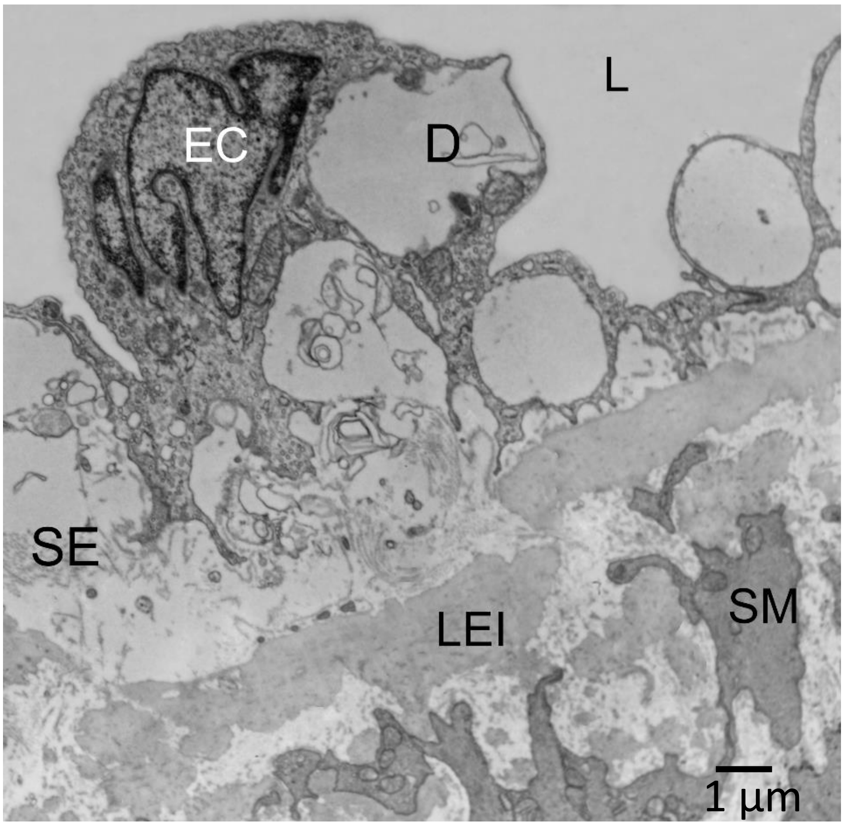

L – lumen of the aorta, EC – endothelial cells with deposits of lipolysis (D), SE – enlarged subendothelial space, LEI – lamina elastica interna, SM – smooth muscle cells, D – deposits of lipolysis. Fixation with gluatraldehyde.
